# Mesenchymal stem cells and their exosomes mitigate osteoarthritis by restoring the balance between proinflammatory Teffs and Tregs

**DOI:** 10.3389/fragi.2024.1509014

**Published:** 2024-11-19

**Authors:** Tianhao Liu, Chunxiao Ran, Dewei Zhao, Fan Yang, Qiang Guo, Jiahui Yang, Xiuzhi Zhang

**Affiliations:** ^1^ Zhongshan Clinical College, Dalian University, Dalian, Liaoning, China; ^2^ Department of Orthopedics, Affiliated Zhongshan Hospital of Dalian University, Dalian, Liaoning, China

**Keywords:** osteoarthritis, regulatory T cell, mesenchymal stem cell, effector T cell, immunomodulatory

## Abstract

Osteoarthritis (OA) is a degenerative joint disease caused by chronic inflammation that damages articular cartilage. In addition to the wear and tear of joints, aberrant remodelling driven by a significant presence of inflammatory mediators within the joint is one of the key mechanisms in the pathogenesis of OA. Among these factors, hyperactivation of Teffs subsets plays a crucial role in promoting this pathological process. The immune imbalance between proinflammatory CD4^+^ effector T cells (proinflammatory Teffs) and Tregs could be a crucial factor in the pathogenesis of OA. Therefore, correcting the imbalance of Tregs/proinflammatory Teffs may slow or inhibit the occurrence and development of OA, which could be a potential target for the treatment of OA. Mesenchymal stem cells (MSCs) possess anti-inflammatory and immunomodulatory properties, regulating both adaptive and innate immunity through mechanisms involving soluble factors such as IDO, PGE2, and TGF-β, as well as cell-to-cell contact and exosomes. Correcting the imbalance between Tregs and proinflammatory Teffs may be one of the mechanisms of MSCs in the treatment of OA. Therefore, this review aims to summarize the relationship between OA and the immune imbalance between Tregs and proinflammatory Teffs, the immunoregulatory role of Tregs in OA, and the role of MSCs and their exosomes in correcting the imbalance between Tregs and proinflammatory Teffs.

## 1 Introduction

Osteoarthritis (OA) is a widespread joint disease worldwide. According to statistical data, approximately 10% of men and 18% of women over the age of 60 suffer from OA, significantly impacting their quality of life. Consequently, OA has become a major healthcare burden in society (Glyn-Jones et al., 2015).

The pathological features of OA include degeneration of articular cartilage, intra-articular inflammation with synovitis, and alterations in the subchondral bone (Chow and Chin, 2020). Research suggests that OA is not merely a degenerative or wear-and-tear disease but also involves abnormal remodelling of joint tissues driven by a significant presence of inflammatory mediators within the joint (Loeser et al., 2012). OA patients often exhibit infiltration of inflammatory cells, such as macrophages, neutrophils, T cells, B cells, natural killer cells, and dendritic cells, into the synovium. Among them, various types of T cells, including T helper (Th) cells, cytotoxic T (CTL) cells, and regulatory T (Treg) cells, make up approximately 22% of the immune cells infiltrating the synovial tissue of OA patients (Li et al., 2017). CD4^+^ T cells can differentiate into effector subsets with different immune functions, such as helper T cells 1 (Th1), Th2, Th9, Th17, Th22, and follicular helper T (Tfh) cells and regulatory T (Treg) cells. These cell subsets drive effects or regulate immune responses by secreting various cytokines. For example, Th1 cells promote inflammation by secreting IL-2, interferon-γ (IFN-γ), and tumor necrosis factor (TNF), whereas Th17 cells produce IL-17. In contrast, Tregs exhibit anti-inflammatory and immunosuppressive functions by secreting IL-10 and transforming growth factor-β (TGF-β). CD4^+^ Teffs subsets comprise two functionally opposing groups: proinflammatory Teffs (such as Th1, Th17, and Th22) and Tregs. The former promotes inflammation, whereas the latter exerts anti-inflammatory effects. The balance between Tregs and proinflammatory Teffs plays a crucial role in maintaining systemic immune homeostasis (Drescher et al., 2020). Researchers have investigated various diseases, such as primary biliary cirrhosis, Alzheimer’s disease, and Parkinson’s disease, from the perspective of the immune imbalance between Tregs and proinflammatory Teffs (Anderson et al., 2014; Drescher et al., 2020). Other studies have also indicated that the onset and progression of OA are closely associated with the immune imbalance between Tregs and pro-inflammatory Teffs (Li et al., 2017). This finding allows the pathogenesis of OA to be integrated into the theoretical framework of immune imbalance, similar to other diseases, providing valuable insights for further in-depth research.

Mesenchymal stem cells (MSCs) not only possess the potential for multi-lineage tissue cell differentiation but also have immunomodulatory functions, allowing them to exert both direct and indirect effects on the adaptive and innate immune systems through multiple mechanisms (Zhang et al., 2024). Firstly, MSCs directly inhibit the activity of T cells and B cells and promote the generation of regulatory T cells by secreting key factors such as IDO, iNOS, and PD-L1, thereby suppressing adaptive immune responses. In the innate immune system, MSCs indirectly inhibit T cell activation through interactions with macrophages and dendritic cells. Particularly in high-inflammatory environments (such as those with high concentrations of IFN-γ and TNF-α), MSCs further enhance their immunosuppressive effects by increasing the expression of iNOS and IDO (Wang et al, 2022b). Additionally, MSCs play a crucial role in enhancing immune tolerance and maintaining tissue homeostasis through their apoptotic processes and the secretion of growth factors, such as HGF and IGF2. Apoptotic MSCs interact with macrophages to induce the release of the immunosuppressive factor IDO, thereby promoting immune tolerance. Other factors secreted by MSCs, such as PGE2, further reduce T cell activity, supporting tissue repair and immune balance (Song et al., 2020). MSCs are considered to have great potential to correct the immune imbalance between Teffs and proinflammatory Tregs because of their unique anti-inflammatory and immunomodulatory properties (Bernardo and Fibbe, 2013). Although the immunomodulatory mechanism of MSCs is complex, it is primarily through cell‒cell contact and the secretion of soluble factors that MSCs mediate their immunomodulatory functions, including inhibiting T-cell and dendritic cell maturation, reducing B-cell activation and proliferation, suppressing natural killer cell proliferation and toxicity, and promoting the generation of Tregs (Sun et al., 2012). These immunomodulatory mechanisms endow MSC therapy with great potential in addressing the chronic inflammation associated with OA.

Therefore, this article reviewed the relationships between Tregs and proinflammatory Teff imbalances and OA, the immunomodulatory effects of Tregs in OA, and the application of MSCs and their exosomes to correct Treg and proinflammatory Teff imbalances in the treatment of OA.

## 2 Imbalance of Tregs and proinflammatory Teffs in osteoarthritis

The pathogenesis of OA is characterized by a persistent low-grade inflammatory process rather than being solely attributed to degeneration or abrasion (Apinun et al., 2016; Fu et al., 2018). This low-grade chronic inflammation is initiated by mechanical wear of cartilage, which subsequently leads to the release of extracellular matrix (ECM) fragments and damage-related molecules and is maintained by the activation of innate and adaptive immune responses (Robinson et al., 2016). Synovitis is one of the main pathological changes in OA (Loeser et al., 2012). Synovitis often occurs in the early stages of OA and after joint injury, and its prevalence increases with the progression of OA (Benito et al., 2005; Krasnokutsky et al., 2011). In a previous study, OA patients who presented with synovitis had more severe baseline cartilage lesions (Ayral et al., 2005). The synovium of OA patients contains many inflammatory immune cells, such as M1 macrophages, Th1 cells and Th17 cells, as well as a small number of Tregs. The relative imbalance between these inflammatory immune cells and their secreted proinflammatory mediators and Tregs may be the internal cause of synovitis (Sohn et al., 2022).

For example, IL-17 secreted by Th17 cells is considered to be a highly relevant factor in the induction of synovitis (Lubberts, 2008). *In vitro* studies have shown that IL-17 can further induce the production of inflammatory cytokines such as TNF-α, IL-1β, IL-6 and matrix metalloproteinases, thereby inducing an inflammatory response (Liu et al, 2015a). In addition, IL-17 can attract more immune cells (such as neutrophils) into the inflammatory site to maintain the inflammatory response (Grieshaber-Bouyer et al., 2019). One study revealed that the Th17 and serum IL-17 levels in OA patients were significantly greater than those in healthy controls (Qi et al., 2016). Another study revealed that the IL-1β, IL-17 and IL-22 levels were elevated in patients with temporomandibular joint OA and were associated with Th1, Th17 and Th22 immune inflammatory response patterns (Monasterio et al., 2018). These studies revealed that an abnormal increase in Teff cells, such as Th1, Th17 and Th22 cells, is closely related to OA.

However, studies have shown that OA patients have a lower percentage of Tregs in the synovium and peripheral blood, which decreases T-cell tolerance and exacerbate inflammation (Moradi et al., 2014). For example, Ponchel et al. (2015) analyzed the blood of 121 healthy individuals and 114 OA patients, reporting that OA patients not only had a lower number of Tregs compared to healthy controls but also exhibited dysfunctional Tregs. Li et al. (2016) reported that Tregs secreted less IL-10 in OA patients, and more advanced OA patients presented a further decrease in IL-10 secretion by Tregs. Lymphocyte activation gene 3 (LAG 3) is a regulator of T-cell survival, activation, proliferation and death. In conventional T cells, LAG-3 functions as an inhibitory receptor that suppresses T-cell inflammatory responses. However, the role of LAG-3 in Tregs remains controversial. Studies have shown that Tregs from OA patients tend to exhibit high expression of LAG-3. Compared with LAG-3- Tregs, LAG-3^+^ Tregs have a reduced capacity for proliferation, cytokine secretion (such as IL-10 and TGF-β), and the inhibition of proinflammatory Teff proliferation. These findings suggest that in OA, Tregs are enriched with subsets highly expressing LAG-3, which limits their anti-inflammatory capabilities. This functional impairment disrupts the immune balance between Tregs and proinflammatory Teffs, ultimately contributing to the onset and progression of OA (Xia et al., 2017). Therefore, there is a close relationship between OA and the Treg and proinflammatory Teff immune imbalance.

In OA, the imbalance between T cells, particularly Tregs and Teffs, plays a critical role in disease progression (Zhang et al., 2024). Th17 cells, as a major subset of proinflammatory Teffs, release cytokines such as IL-17, which stimulate inflammatory mediators, exacerbating local joint inflammation and accelerating cartilage degradation. In contrast, Tregs suppress immune responses through the secretion of anti-inflammatory cytokines, such as IL-10, maintaining immune homeostasis within the joint. However, in OA patients, an increase in Th17 cells and weakened Treg function lead to a Th17/Treg imbalance, where proinflammatory responses predominate, thus driving OA pathogenesis (Wen et al., 2024). Furthermore, the imbalance between Tregs and Teffs, particularly Th17 cells, directly affects bone metabolism. Th17 cells promote osteoclast activity, accelerating bone resorption, whereas Tregs can inhibit this process, thereby preserving joint integrity (Zhu et al., 2020). Studies suggest that modulating the Th17/Treg ratio can effectively suppress joint inflammation and slow cartilage degradation, highlighting the therapeutic significance of maintaining Th17/Treg balance in OA. Targeted modulation of the Th17/Treg imbalance, through approaches such as anti-IL-17 antibodies or strategies that enhance Treg function, holds promise as a viable therapeutic avenue for OA in the future (Wan et al, 2022a).

**FIGURE 1:**
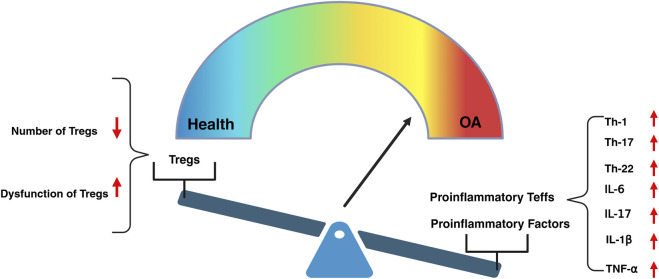
The decline in the number and dysfunction of Tregs, leading to the excessive activation of proinflammatory Teffs, serves as a driving force in the initiation and progression of OA. The excessive activation of proinflammatory Teffs, such as Th-1, Th-17and Th-22, leads to the upregulation of pro-inflammatory factors, including IL-6,IL-17, IL-1β, and TNF-α, which triggers inflammatory responses within the joint tissues.

Recent studies have demonstrated that the Notch signaling pathway plays a key role in regulating the immune balance between proinflammatory Teffs and Tregs. Th17 cells are a key subset of proinflammatory effector T cells, playing an important role in immune responses, particularly in anti-infection and autoimmune diseases (Zhang et al., 2024). Th17 cells primarily mediate inflammatory responses by secreting cytokines such as IL-17, IL-21, and IL-22, which stimulate the activation of other immune cells and the production of inflammatory mediators. Li et al. (2018) confirmed that Notch1 mRNA expression is positively correlated with the Th17/Treg ratio. During the inflammatory response, when Notch1 signaling is enhanced, the expression of RORγt significantly increases, while the expression of Foxp3 significantly decreases, thereby regulating the differentiation of Th17 cells and Treg cells. Furthermore, Brisslert et al. (2014) further discovered that blocking Notch signaling with DAPT (a γ-secretase inhibitor) can significantly inhibit the differentiation of Th17 cells and reduce the number of Th17 lineage cells. Sun et al. (2018) found in an OA animal model that the use of Jagged1 to inhibit the Notch signaling pathway significantly enhances cartilage formation in OA mice and reduces the expression of inflammatory mediators such as TNF-α, IL-1β, and MMP13 in synovial tissue, thereby effectively alleviating inflammatory responses in the joints, including a reduction in chondrocyte apoptosis and an increase in the expression of type II collagen. By reversing the decrease in the number and function of Treg subsets, it may be possible to correct the immune imbalance between Tregs and proinflammatory Teffs, thereby reshaping the reparative immune microenvironment and facilitating the mitigation or even prevention of OA (Wen et al., 2024) (Figure 1).

## 3 The immunoregulatory effect of Tregs in OA

The regulatory T cell (Treg) family is composed of several heterogeneous subsets, including CD4, CD8, natural killer T (NKT) cells, and γδ T cells. Among these Treg subsets, CD4^+^ Treg plays a crucial role in regulating immune balance, protecting the body from excessive immune responses, and promoting self-repair (Savage et al., 2020; Zhang et al., 2023). Nees et al. (2022) found that the number of Tregs is significantly reduced in patients with OA, leading to a decrease in the release of anti-inflammatory mediators such as IL-10, which exacerbates joint inflammation. Therefore, studying the role of Tregs in OA not only aids in elucidating the pathological mechanisms but also provides important targets for the development of new immunotherapeutic strategies, highlighting their significant clinical application potential.

### 3.1 Classification of Treg subsets and immune balance

CD4^+^ Treg subsets are categorized into two types on the basis of their site of development: natural regulatory T cells (nTregs) and induced regulatory T cells (iTregs). nTregs, also known as CD4^+^CD25^+^Foxp3^+^ T cells, develop in the thymus and exhibit high heterogeneity (Liu et al, 2015b). iTregs are generated from CD4^+^ T cells in peripheral immune organs in response to antigen stimulation and are primarily classified into two types: Tr1 (type 1 regulatory T cells), which produce IL-10, and Th3 (T helper 3 cells), which produce TGF-β (Chien and Chiang, 2017). nTregs and iTregs maintain peripheral immune tolerance by suppressing potential autoimmune responses while also controlling excessive proinflammatory Teff responses to infections, thereby achieving a proper immune balance.

### 3.2 Immunomodulatory role of nTregs

**FIGURE 2:**
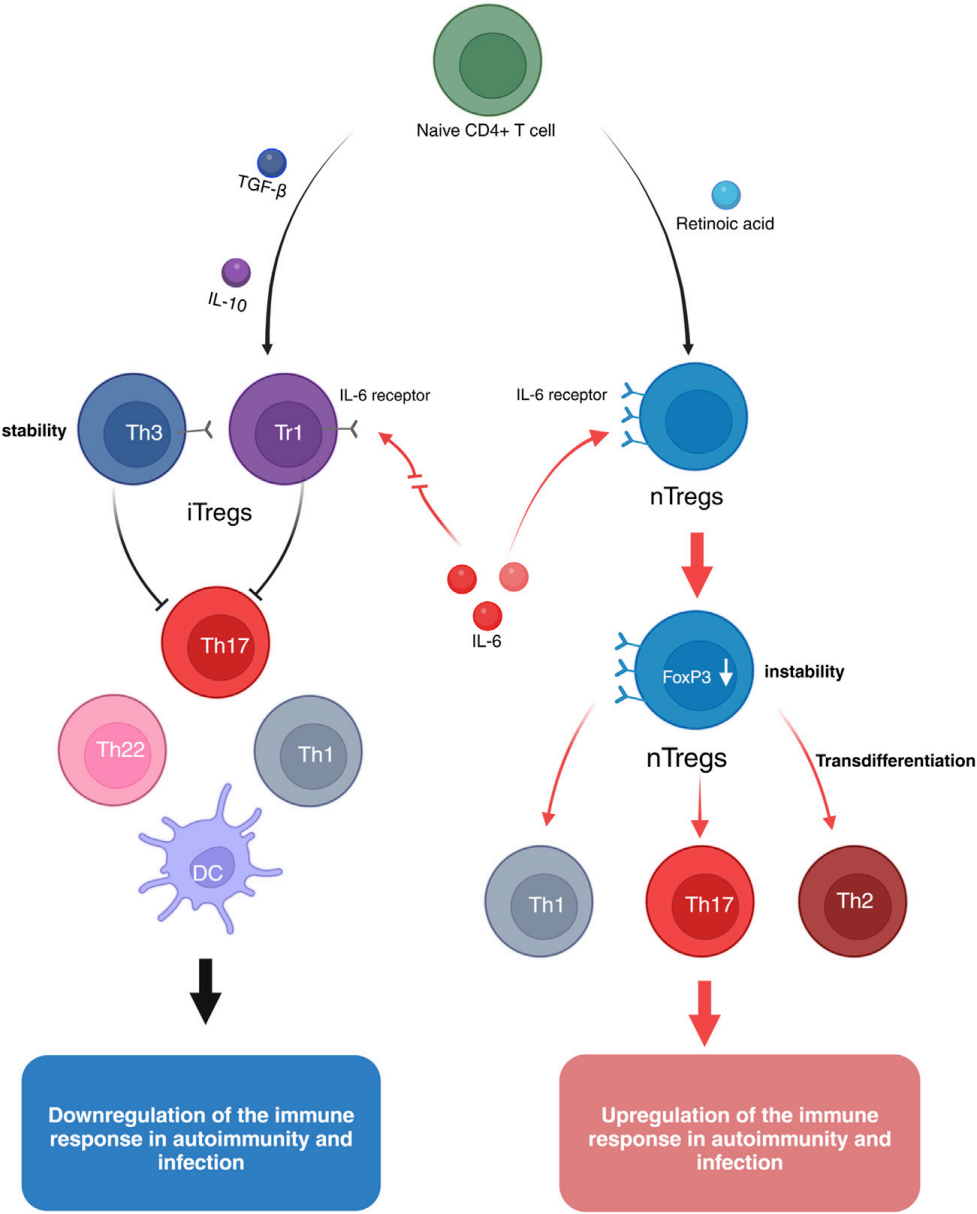
Naive CD4^+^ T cells can be induced to differentiate into natural regulatory T cells (nTregs) by retinoic acid. nTregs express high levels of the IL-6 receptor, and in the presence of pro-inflammatory cytokines such as IL-12, IFN-γ, and IL-6, Foxp3 expression is reduced, leading to the transdifferentiation into Th17, Th1, and Th2 phenotypes, thus exhibiting instability. Induced regulatory T cells (iTregs) can be generated from naive CD4^+^ T cells through the induction by IL-10 or TGF-β. IL-10-induced Tr1 cells or TGF-β-induced Th3 cells express low levels of the IL-6 receptor. Even in the presence of pro-inflammatory cytokines like IL-6, iTregs maintain their suppressive function and remain stable.

Natural regulatory T cells (nTregs) originate from progenitor cells in the bone marrow, undergoing lineage differentiation and maturation in the thymus. nTregs continuously express the transcription factor Foxp3, which is closely associated with their development and function, serving as a key regulatory factor for nTregs (Raffin et al., 2020). Research has shown that the injection of anti-CD25 monoclonal antibodies depletes the majority of nTregs, significantly increasing the incidence of arthritis in mice, whereas the transplantation of nTregs can reverse the progression of arthritis (Zhou et al., 2011). Furthermore, H. Kelchtermans’ study also demonstrated that the transplantation of purified nTregs into arthritic mice can inhibit disease progression (Kelchtermans et al., 2009). However, the therapeutic application of nTregs is limited by their instability. In the presence of IL-6, nTregs exhibit reduced stability and are prone to differentiate into Th17 cells, which secrete IL-17 a cytokine closely associated with joint inflammation in arthritis (Lubberts, 2008). IL-17 can also induce the production of various pro-inflammatory cytokines, thereby exacerbating inflammation (Liu et al, 2015b). Therefore, the intrinsic instability of nTregs may limit their capacity to effectively control the progression of chronic inflammation, wherein IL-6 plays a pivotal role in modulating their stability and functional integrity. Targeting the IL-6 signaling pathway may thus represent a promising therapeutic strategy for harnessing nTregs in the management of chronic immune-mediated inflammatory conditions (Figure 2).

### 3.3 Immunomodulatory effects of iTregs

Induced regulatory T cells (iTregs) are a subset of regulatory T cells formed through peripheral exposure to antigens or stimulation by specific cytokines. Unlike natural regulatory T cells (nTregs), iTregs primarily develop in peripheral tissues and can be induced by cytokines such as IL-10 and TGF-β. Compared to nTregs, iTregs exhibit a more stable anti-inflammatory capacity during immune responses, which helps prevent excessive inflammation and the onset of autoimmune diseases (Chen et al., 2021; Kanamori et al., 2016). For example, experiments by Ning Kong et al. demonstrated that, *in vitro*, iTregs possess a stronger ability than nTregs to suppress proinflammatory Teffs and mitigate disease progression in arthritic mice (Kong et al., 2012). However, the significant therapeutic effects of iTregs on arthritis may be attributed to their expression of lower levels of IL-6 receptors (Mohan et al., 2023; Zheng et al., 2008). Therefore, these cells exhibit resistance to IL-6 stimulation and are capable of inhibiting the differentiation of Th17 cells induced by IL-6 and TGF-β. This characteristic may contribute to enhancing the stability and suppressive function of iTregs, thereby improving their therapeutic efficacy (Kong et al., 2012) (Figure 2).

### 3.4 Mechanisms of Treg-mediated suppression of Teffs

**FIGURE 3:**
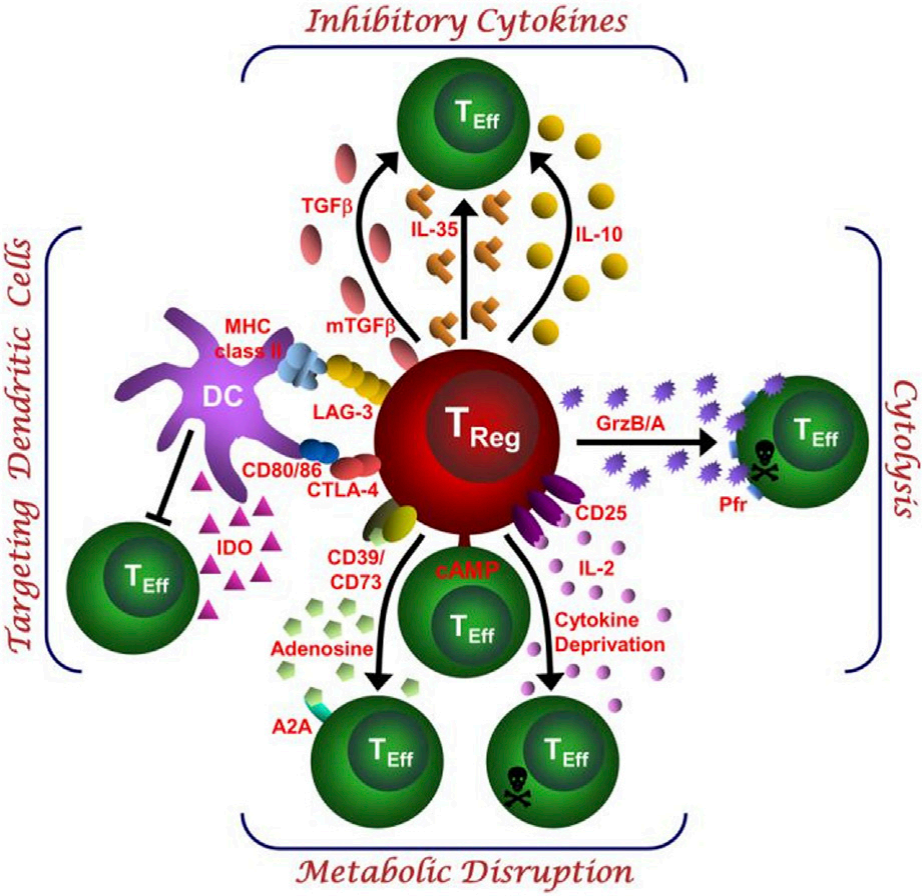
This schematic depicts the various regulatory T (Treg)-cell mechanisms arranged into four groups centred around four basic modes of action. “Inhibitory cytokines” include interleukin-10 (IL-10), interleukin-35 (IL-35) and transforming growth factor-β (TGF-β). “Cytolysis” includes granzyme-A- and granzyme-B-dependent and perforin-dependent killing mechanisms. “Metabolic disruption” includes high affinity IL-2 receptor α (CD25)-dependent cytokine-deprivation-mediated apoptosis, cyclic AMP (cAMP)-mediated inhibition, and CD39^−^and/or CD73-generated, adenosine–purinergic adenosine receptor (A2A)-mediated immunosuppression. “Targeting dendritic cells” includes mechanisms that modulate DC maturation and/or function such as lymphocyte activation gene-3 (LAG3; also known as CD223)–MHC-class-II-mediated suppression of DC maturation, and cytotoxic T lymphocyte antigen-4 (CTLA4)–CD80/CD86-mediated induction of indoleamine 2,3-dioxygenase (IDO), which is an immunosuppressive molecule, by DCs (Vignali et al., 2008).

Tregs broadly suppress proinflammatory Teffs, such as Th1, Th17, and Th22 T cells, through various mechanisms, including cytokine production, cytolysis, metabolic disruption, and the regulation of dendritic cell (DC) maturation or function (Boonpiyathad et al., 2020; Vignali et al., 2008) (Figure 3).

#### 3.4.1 Suppressing Teffs by anti-inflammatory factors

The paracrine secretion of anti-inflammatory cytokines, such as IL-10 and TGF-β, by Tregs is a key mechanism by which Tregs inhibit the proinflammatory activation of Teffs. Tregs suppress the global protein synthesis of proinflammatory Teffs by specifically inhibiting mRNAs of the translation machinery at the level of mTORC1-mediated translation control through the concerted action of the immunosuppressive cytokines IL-10 and TGF-β (So et al., 2023). Moreover, Tregs effectively suppress the proliferation and function of proinflammatory Teffs by secreting IL-35, thereby maintaining immune homeostasis. IL-35 is composed of the EBI3 and P35 subunits in Tregs and functions independently of Foxp3 expression, exerting its suppressive effect remotely, even under non-contact conditions. This mechanism enables Tregs to downregulate proinflammatory Teffs activity in inflammatory environments, helping to prevent excessive immune responses and suppress autoimmune diseases (Yang et al., 2024).

#### 3.4.2 Suppressing Teffs by cytolysis

Other studies have shown that Tregs mediate the lysis of proinflammatory Teffs by expressing granzyme B and perforin, which inhibits proinflammatory Teff activation through cytotoxic pathways (Arce-Sillas et al., 2016). Additionally, Tregs induce the apoptosis of proinflammatory Teffs via the TRAIL–DR5 (tumor necrosis factor-related apoptosis-inducing ligand-death receptor 5) pathway, thereby enhancing their anti-proliferative and anti-inflammatory effects (Cassano et al., 2023).

#### 3.4.3 Suppressing Teffs by targeting dendritic cells

Tregs also indirectly regulate the maturation or function of dendritic cells (DCs) to inhibit the activation of proinflammatory Teffs. For example, Tregs impair the T-cell-stimulating capacity of DCs by downregulating the costimulatory molecules CD80, CD86, and CD40 and reducing the levels of MHC-peptide complexes on DCs while upregulating the inhibitory B7-H3 molecule, a member of the programmed death ligand family (Zong et al., 2024). Moreover, Tregs have been shown to significantly inhibit the secretion of proinflammatory cytokines such as IL-12, IL-1β, IL-6, and IL-8 by dendritic cells (DCs) while increasing the expression of the anti-inflammatory cytokine IL-10 (Andre et al., 2009; Zhang et al., 2020). Through the interaction of CTLA4 with CD80/CD86, Tregs induce the expression of indoleamine 2,3-dioxygenase (IDO), an enzyme that catalyses the conversion of tryptophan into the immunosuppressive metabolite kynurenine, in dendritic cells. This depletes tryptophan, which is essential for the growth of proinflammatory Teffs in their microenvironment, thereby inhibiting their proliferation (Lippens et al., 2016).

#### 3.4.4 Suppressing Teffs by metabolic signal

Several intriguing suppressive mechanisms of Tregs have been reported, which can collectively be referred to as suppression by metabolic disruption pathways. Studies have shown that Tregs generate extracellular adenosine through the expression of CD39 and CD73, which activate A2A receptors on proinflammatory Teffs, thereby inhibiting IL-6 expression and promoting TGF-β secretion. This ultimately suppresses the function of proinflammatory Teffs, such as Th17 cells, while promoting the generation of Tregs (Kobie et al., 2006; Zarek et al., 2008). Moreover, Tregs can transfer the potent inhibitory second messenger cyclic adenosine monophosphate (cAMP) to proinflammatory Teffs through gap junctions, thereby directly suppressing the function of pro-inflammatory Teffs (Bopp et al., 2007). Thornton et al. suggested that Tregs can “deplete” local IL-2 by highly expressing CD25, thereby inhibiting the proliferation of proinflammatory Teffs by depriving them of the IL-2 necessary for their survival (Arce-Sillas et al., 2016). Although these mechanisms provide intriguing insights into the immunoregulatory roles of Tregs, further research is needed to validate these significant findings and explore the specific contributions of these mechanisms to the functions of Tregs, as well as their potential applications.

## 4 The correction of Treg imbalance for the treatment of OA

At present, treatment methods for early- or mid-stage OA, such as nonsteroidal anti-inflammatory drugs, intra-articular injection of sodium hyaluronate, and arthroplasty, can only relieve symptoms and improve function and cannot block the progression of the disease. OA patients with severe dysfunction can only choose surgical arthroplasty. However, this treatment may cause complications such as deep vein thrombosis, joint infection, prosthesis loosening or fracture after surgery. Furthermore, owing to the limited lifespan of prosthetics, younger patients may require multiple revision surgeries, which not only increases medical costs but also imposes a physical and psychological burden on the patients (Billesberger et al., 2020). Therefore, there is an urgent clinical need for a treatment method that can reverse the progression of OA in the early stage of OA to avoid patients being forced to choose joint replacement. With the development of medical immunology and the in-depth study of the immune mechanism of OA, the intervention of Treg subsets may be considered a new strategy for the treatment of OA.

### 4.1 Mesenchymal stem cells improve OA by correcting Treg and proinflammatory Teff imbalances

**FIGURE 4:**
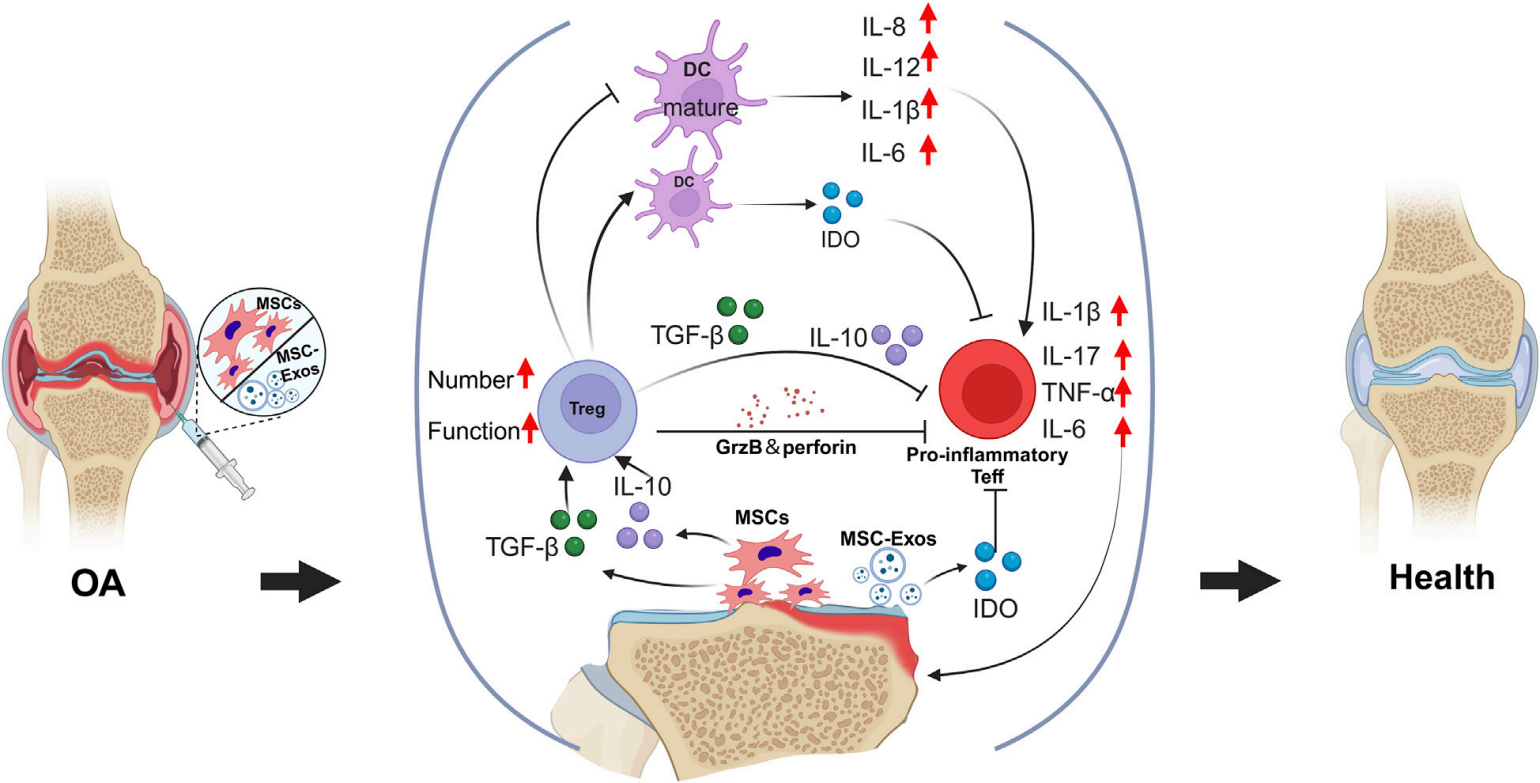
The transplantation of MSCs and their exosomes facilitates the correction of the imbalance between pro-inflammatory and anti-inflammatory responses within the osteoarthritic tissue microenvironment. Through immunoregulatory paracrine signaling, MSCs enhance both the quantity and functionality of Tregs, which, in turn, suppress the excessive activity of pro-inflammatory Teffs.

OA is widely recognized as a chronic low-grade inflammatory disease in which various immune cells play critical roles in its pathogenesis and progression (Li et al., 2017). An imbalance between Th17 cells and Tregs has a significant effect on the pathogenesis of OA. Th17 cells primarily secrete IL-17, disrupting the homeostasis of the extracellular matrix and serving as a key mediator in the pathogenesis of chronic inflammatory diseases. In OA, IL-17 further promotes the production of inflammatory cytokines and recruits additional immune cells, such as neutrophils, to the site of inflammation, thereby exacerbating joint damage (Grieshaber-Bouyer et al., 2019). Currently, MSCs are recognized by researchers both domestically and internationally for their ability to correct immune imbalances by modulating various immune cells, thereby inhibiting or slowing the onset and progression of autoimmune diseases (Griffin et al., 2013). Furthermore, numerous studies have demonstrated that MSCs possess significant potential in the treatment of OA (Figure 4).

MSCs are an ideal novel therapeutic strategy because of their unique anti-inflammatory and immunoregulatory properties. Research indicates that MSCs mediate various cellular immune responses through cell‒cell contact and the secretion of soluble factors. These responses include the inhibition of proinflammatory T-cell and dendritic cell maturation, a reduction in B-cell activation and proliferation, the suppression of natural killer cell proliferation and cytotoxicity, and the promotion of Treg generation. These mechanisms are considered one of the primary pathways through which MSCs perform their immunoregulatory functions (Sun et al., 2012). For example, Han et al. (2011) reported that bone marrow-derived MSCs employ a cell contact-dependent mechanism that not only reduces the survival and proliferation of proinflammatory Teffs but also increases the proportion of Tregs. Additionally, research indicates that certain proinflammatory cytokines, such as IFN-γ, TNF-α, IL-1α, and IL-1β, can induce MSCs to secrete various enzymes and soluble factors, including cyclooxygenase-2 (COX-2), prostaglandin E2 (PGE2), and indoleamine 2,3-dioxygenase (IDO), thereby mediating their immunosuppressive activities (DelaRosa et al., 2009).

MSCs can inhibit the differentiation of naive T cells into Th17 cells through the action of prostaglandin E2 (PGE2) and suppress the production of IL-17, IL-22, IFN-γ, and TNF-α by fully differentiated Th17 cells. Interestingly, MSCs can also inhibit the function of Th17 cells by suppressing the expression of retinoic acid receptor-related orphan receptor gamma t (RORγt) and induce the expression of the Treg-associated phenotype Foxp3, as well as the secretion of IL-10, thereby converting Th17 cells into a T-cell population with anti-inflammatory properties (Ghannam et al., 2010). Furthermore, when MSCs are exposed to high levels of proinflammatory cytokines, they directly induce the generation of Tregs by secreting soluble factors such as indoleamine 2,3-dioxygenase (IDO), prostaglandin E2 (PGE2), and transforming growth factor-beta (TGF-β) while also promoting the polarization of macrophages into M2-type macrophages. Moreover, M2 macrophages secrete substantial amounts of IL-10 and CCL18, further enhancing the generation of Tregs (Bernardo and Fibbe, 2013; Melief et al., 2013). Therefore, MSCs have the potential to serve as a therapeutic strategy for OA by reversing the imbalance between Th17 cells and Tregs through the suppression of Th17 cell numbers and function while promoting the quantity and functionality of Tregs.

However, several limitations are associated with the use of MSC transplantation for the treatment of OA. For example, autologous stem cells require an extended period of *in vitro* expansion prior to transplantation, and their viability and functionality are susceptible to the effects of donor age, underlying diseases, and genetic heterogeneity. Allogeneic stem cell transplantation, on the other hand, faces challenges related to immunogenicity and ethical considerations. Moreover, the stemness and functional stability of MSCs derived from different sources, such as bone marrow, adipose tissue, and the umbilical cord, are variable. During application, the effective components released by MSCs are diverse and dependent on the host microenvironment, including factors such as inflammatory status, hypoxia, and the extracellular matrix (ECM), which contributes to the high adaptive heterogeneity in MSC (Zhou et al., 2021).

MSCs exhibit significant heterogeneity influenced by factors such as donor age, metabolic state, and genetic background, complicating the evaluation of their therapeutic safety and efficacy and limiting advancements in standardized and personalized MSC therapies (Zhang et al., 2024). For instance, in systemic diseases such as lupus, diabetes, and rheumatoid arthritis, allogeneic MSC transplantation has shown greater efficacy than autologous MSC transplantation (Costa et al., 2021). The therapeutic efficacy of autologous MSCs in OA is also limited by genetic background factors, such as abnormal GDF5 expression, which can impair their proliferation and chondrogenic differentiation capacity (Wang et al., 2020). Given that OA is more prevalent among elderly patients with metabolic disorders, it is crucial to address MSC heterogeneity in stem cell therapies for OA (Vina and Kwoh, 2018; Zhang et al., 2024). Among various MSC sources, Wharton’s jelly-derived stem cells (WJSCs) are particularly noteworthy for their relative homogeneity. WJSCs have comparatively long telomere lengths, low immunogenicity, broad differentiation potential, high proliferative capacity, and lack of tumorigenicity. Additionally, the extracellular matrix of WJSCs is rich in hyaluronic acid, sulfated glycosaminoglycans, and collagen, closely resembling the matrix of chondrocytes. This similarity is advantageous for cartilage repair. Therefore, by employing genetic sequencing to minimize heterogeneity from genetic backgrounds, WJSCs may represent the most suitable homogeneous MSC source for stem cell therapy in osteoarthritis (Matas et al., 2019).

OA is not merely a result of joint wear and tear; rather, it is a complex immune-mediated disease. While MSCs were initially employed in OA treatment for their potential in cartilage regeneration—demonstrating significant efficacy through injection and transplantation current evidence suggests that MSCs exert their primary effects in OA through immunomodulatory and anti-inflammatory functions rather than direct cartilage regeneration (Matas et al., 2019). In the inflammation-driven OA environment, MSCs release cytokines and exosomes that modulate immune responses, establishing a regenerative-supportive microenvironment. These factors help balance Treg and Th17 cells, as well as M1 and M2 macrophages, contributing to OA progression inhibition and symptom relief. In the short term, MSCs primarily achieve therapeutic effects by suppressing inflammation and enhancing the immune microenvironment rather than by direct cartilage repair. Over time, MSCs may facilitate partial cartilage defect repair through microenvironmental improvements, achieving sustained symptom relief and disease control (Zhang et al., 2024). Moreover, both donor and recipient age significantly influence the functionality and therapeutic efficacy of MSCs (Chahal et al., 2019). Autologous MSCs (such as BMSCs and ADMSCs) and allogeneic WJSCs from younger donors can effectively decelerate cartilage degeneration in younger OA patients, potentially reducing the need for joint replacement therapy (Lu et al., 2019; Matas et al., 2019). For elderly patients, allogeneic MSCs from younger donors are preferable, as they exhibit markedly superior anti-inflammatory and immunomodulatory properties compared to autologous MSCs (Matas et al., 2019). Consequently, an age-specific donor-recipient strategy could enhance MSC therapy, yielding improved therapeutic outcomes for OA treatment.

### 4.2 MSC-exos improve OA by correcting Tregs and pro-inflammatory Teffs imbalance

Currently, in some countries and regions, stem cell therapies face strict ethical restrictions. As an alternative, the transplantation of stem cell-derived exosomes has gained extensive research interest and application. Due to its close relationship with stem cell transplantation in terms of origin and mechanism, this approach has attracted considerable attention in the field of OA treatment. Studies have shown that MSC-derived exosomes can promote cartilage repair and regeneration in OA through various mechanisms, including enhanced matrix synthesis, modulation of anti-inflammatory immune responses, and inhibition of apoptosis (Nguyen et al., 2021; Zhang et al., 2024). Exosomes derived from mesenchymal stem cells (MSC-Exos) are small extracellular bilayer membrane vesicles with a diameter of 30–100 nm that are secreted via paracrine mechanisms. They facilitate biological responses in recipient cells by transferring a variety of signalling molecules, including proteins, cytokines, microRNAs, and mRNAs, between cells. Therefore, MSC-Exos are considered important tools for intercellular communication (Kourembanas, 2015). The application of MSC-Exos not only overcomes certain limitations of MSC transplantation, such as heterogeneity, ethical concerns, and the time required for MSCs to migrate to the site of injury but also allows for standardized production of exosomes, along with easier storage and transportation. As the isolation and purification techniques for MSC-Exos continue to improve, they may emerge as novel cell-free therapeutic approaches for the treatment of OA.

MSC-Exos promote chondrocyte proliferation, inhibit chondrocyte apoptosis, reduce proinflammatory cytokines, modulate immune responses, and facilitate the redeposition of the cartilage matrix (Luo et al., 2024). MSC-Exos derived from various types of MSCs have been utilized in cell and animal experiments related to OA (Table 1).

**TABLE 1:** The impact of exosomes sourced from various types of mesenchymal stem cells on OA.

Exosome source	Cargo	Biological effect	References
B-MSC	lncRNALYRM4-AS1	Regulate chondrocyte growth and reduce inflammation in OA	Wang et al (2021a)
	miR-9-5p	Reduce IL-1 and TNF-α in OA by inhibiting SDC1 expression	Jin et al. (2020)
	ND	Promote chondrocyte viability, proliferation, migration by optimizing mitochondrial function	Yang et al. (2022)
S-MSC	miR-155-5p	Promote the proliferation and migration of chondrocytes, reduce the apoptosis of chondrocytes, regulate the extracellular matrix of chondrocytes	Wang et al. (2021b)
WJ-MSC	ND	Promote chondrocyte proliferation	Jiang et al. (2021)
IPFP-MSC	miR -100-5p	Protect articular cartilage by inhibiting mTOR	Wu et al. (2019)
UC-MSC	miR-486-5p, miR-148a-3p	Promote macrophage polarization to the M2 phenotype macrophage, alleviating OA progression	Li et al. (2022)

MSC, mesenchymal stem cells; OA, osteoarthritis; N.D., no data; B-MSCs, bone marrow derived MSCs; UC-MSCs, umbilical cord derived MSCs; S-MSCs, synovial MSCs; IPFP-MSCs, infrapatellar fat pad derived MSCs; SDC1, syndecan-1; mTOR, mammalian target of rapamycin.

A study revealed that exosomes derived from bone marrow mesenchymal stem cells (BMSC-Exos) influence chondrocyte viability, proliferation, and migration by increasing mitochondrial activity (Yang et al., 2022). Wang et al. (2021a) reported that BMSC-Exos deliver the lncRNA LYRM4-AS1, which regulates chondrocyte viability via the LYRM4-AS1/GRPR/miR-6515-5p axis (Wang et al, 2021a). Additionally, miR-9-5p from BMSC-derived exosomes has been shown to suppress SDC1 expression, further reducing IL-1 and TNF-α levels in OA (Jin et al., 2020).

Exosomes derived from miR-155-5p-overexpressing synovial mesenchymal stem cells (SMSC-Exos) have been shown to prevent OA by promoting proliferation and migration, reducing apoptosis, and regulating the extracellular matrix of chondrocytes (Wang et al, 2021b). Another study confirmed that exosomes derived from human umbilical cord Wharton’s jelly MSCs (WJMSC-Exos) can promote chondrocyte proliferation in a dose-dependent manner (Jiang et al., 2021). Exosomes rich in miR-100-5p derived from infrapatellar fat pad (IPFP) MSCs (IPFPMSC-Exos) protect articular cartilage and improve gait abnormalities in OA patients by inhibiting mTOR (Wu et al., 2019). Additionally, UCMSC-derived exosomes (UCMSC-Exos) containing miR-100-5p, miR-let-7a-5p, miR-122-5p, miR-486-5p, and miR-148a-3p mitigate the progression of OA by promoting the polarization of macrophages towards the M2 phenotype (Li et al., 2022).

Zhang et al. (2019) revealed that MSC-derived exosomes (MSC-Exos) inhibit pain and inflammation in the early stages of OA, reduce cartilage degeneration, and subsequently improve cartilage matrix expression and subchondral bone remodelling, ultimately promoting overall joint restoration and regeneration. The immunomodulatory capabilities of MSC-derived exosomes (MSC-Exos) are critical for the early stages of this repair process. Research has indicated that mouse-derived bone marrow mesenchymal stem cell exosomes (BMSC-Exos) can suppress the proliferation of syngeneic or allogeneic T lymphocytes by increasing the proportion of CD4^+^ CD25^+^ Foxp3^+^ regulatory T cells, as well as enhancing the secretion of IL-10 and TGF-β (Mokarizadeh et al., 2012). It has been reported that human umbilical cord-derived exosomes (hUC-Exos) can affect the distribution of T-cell subsets, specifically increasing the number of Tregs while reducing the number of Th17 cells (Guo et al., 2019).

## 5 Discussion and prospect

Osteoarthritis is a chronic degenerative disease, beginning with cartilage degeneration, leading to subchondral bone sclerosis, osteophyte formation, and synovitis. This progression ultimately results in joint structural damage and deformity, accompanied by inflammation and matrix degradation, causing gradual loss of joint function, persistent pain, and limited mobility (Martel-Pelletier et al., 2016; Motta et al., 2023). A study involving 30 osteoarthritis patients showed that 65% of the patients exhibited T-cell aggregation in the synovium, suggesting that abnormally active T cells drive abnormal remodeling of joint tissue (Sakkas et al., 1998). Synovitis is considered a key pathological process in OA, where the abnormal accumulation and imbalance in the ratios of T cells and their subtypes (such as Th1, Th2, Th17 cells, and immunosuppressive iTreg cells like Th3 and Tr1 cells) play a critical role in the formation of synovial inflammation, marking a notable feature of synovitis in OA patients (de Lange-Brokaar et al., 2012). These findings suggest that the dynamic balance of different T-cell subsets is closely related to the occurrence and progression of OA.

This review describes the close relationship between OA and the immune imbalance between Tregs and proinflammatory Teffs. This highlights the instability and even reversibility of Tregs in OA treatment while emphasizing their potential therapeutic role in OA management. The discussion regarding the inhibitory or promotional functions of these Tregs in OA must focus on the local microenvironment and their origins. These factors determine the phenotype and function of Tregs, as well as the secretion of proinflammatory and anti-inflammatory cytokines (Pandiyan et al., 2011). Therefore, a comprehensive understanding of the behaviours and characteristics of different subsets of Tregs in response to various environmental signals is crucial for the effective therapeutic application of these cells.

Existing clinical reports indicate that the transplantation of MSCs can significantly suppress chronic inflammation, alleviate pain, prevent the progression of OA, and demonstrate a degree of cartilage repair effects during long-term treatment. Although the chondrogenic potential of MSCs is highly anticipated in the treatment of OA, MSC transplantation has not demonstrated ideal cartilage regeneration capabilities in the short term. This may be because the *in vivo* microenvironment does not fully support the chondrogenic differentiation of MSCs. Although MSCs can differentiate into chondrocytes under specific culture conditions and growth factor stimulation *in vitro*, the complex inflammatory and immune environment *in vivo*, particularly in the joints of OA patients, may limit the differentiation potential of MSCs (Peng et al., 2023). However, the primary mechanism of MSC transplantation in the early stages of OA treatment involves the immunoregulatory and anti-inflammatory properties of MSCs, which help establish a regenerative microenvironment in damaged tissues (Colombini et al., 2019).

MSCs are aptly referred to as “sensors and regulators of inflammation.” These findings emphasize that MSCs can both sense initial inflammatory signals (acting as sensors) and modulate the intensity and duration of the inflammatory response (acting as regulators) (Bernardo and Fibbe, 2013). During the chronic inflammatory stage of OA, MSCs may be able to sense the dynamic changes in inflammatory cells (such as Tregs and proinflammatory Teffs) and the inflammatory microenvironment in the joint synovium or other tissues. Consequently, they can exert their immunoregulatory effects through the paracrine secretion of various cytokines, such as PGE2 and IDO, or through direct cell contact, thereby correcting the imbalance between Tregs and proinflammatory Teffs, reversing the deteriorated microenvironment, and ultimately achieving therapeutic effects in OA. Therefore, the therapeutic efficacy of MSCs largely depends on the local inflammatory status of the host. These findings indicate that the timing, frequency, and route of MSC transplantation may be critical factors determining therapeutic outcomes in patients with OA.

In OA treatment, MSCs primarily function by establishing a regenerative microenvironment within damaged tissues, suppressing chronic inflammation and disease progression, thereby delaying or potentially avoiding joint replacement (Colombini et al., 2019). While MSCs are anticipated to possess cartilage differentiation potential, the *in vivo* microenvironment particularly the complex inflammatory and immune environment within OA-affected joints may not fully support MSC cartilage differentiation, potentially limiting their regenerative capacity. As a result, MSC transplantation can effectively slow OA progression but is not yet sufficient to replace joint replacement surgery (Peng et al., 2023). Future approaches may include tissue engineering strategies to further enhance MSC cartilage differentiation and regeneration, providing a more comprehensive OA treatment strategy. For example, biodegradable scaffolds can offer 3D structural support for MSCs, while the addition of specific growth factors (e.g., TGF-β) can promote their differentiation into chondrocytes. Additionally, physical (e.g., mechanical stretching) and chemical (e.g., hypoxic conditions) stimuli may further boost MSC cartilage differentiation efficiency. Furthermore, MSC cell count, source, injection timing, and treatment frequency are key factors influencing *in vivo* cartilage differentiation (Mamachan et al., 2024). Although MSC transplantation holds promise as a standard OA treatment, its application still faces significant challenges. Foremost, the lack of large-scale, long-term follow-up data limits validation of its efficacy and long-term safety. Additionally, stringent ethical and regulatory restrictions have hindered the clinical advancement of MSCs, while the high costs of production and application, coupled with technical challenges in ensuring batch-to-batch consistency in cell quality and efficacy, further restrict its development. Together, these factors contribute to MSCs not yet being established as a standard clinical treatment for OA (Arshi et al., 2020).

In terms of their paracrine therapeutic mechanisms and effects, MSCs serve as drug delivery vehicles for localized inflammatory regions. Consequently, novel molecular tools rich in the MSC secretome, proteome, and transcriptome such as MSC-derived exosomes (MSC-Exos) may replace MSC-based cell therapies (Biancone et al., 2012). However, the mechanism by which MSCs correct the immune imbalance between Tregs and proinflammatory Teffs is partially mediated by the synergistic interactions of various molecules within their secretome. Therefore, it is essential to consider this complexity when designing and utilizing MSC-derived microvesicles or exosomes for the treatment of OA.

Furthermore, the application of MSC-Exos is hindered by several limitations, including low yield, poor circulation stability, and insufficient targeting ability, which restrict their therapeutic efficacy in the treatment of OA (Kimiz-Gebologlu and Oncel, 2022). To overcome these challenges and advance the clinical application of exosome therapy, engineered exosomes have emerged as a hotspot research area. Currently, various engineering methods have been developed, including cargo loading, surface modification, alteration of production environments, and combinations of biomaterials. These exosomes not only have the potential to overcome the uncertainties associated with the cellular heterogeneity of MSC therapies but also enhance the targeting efficacy of personalized OA treatment (Cheng et al., 2022). However, MSC-Exos contain various functional proteins and immunomodulatory molecules, which may provoke a strong response from the host immune system upon the use of engineered exosomes, leading to their rapid clearance. Moreover, various factors, such as storage conditions and duration, administration routes, and dosage, can significantly influence the bioactivity and therapeutic efficacy of MSC-Exos (Luo et al., 2024). Promisingly, clinical studies have been conducted to evaluate the efficacy of MSC-Exos in treating OA, with the results indicating that MSC-Exos therapy is safe and effective, potentially serving as an alternative to joint replacement surgery or MSC transplantation.

## 6 Conclusion

Helper T (Th) cells play crucial roles in the occurrence and progression of OA. Proinflammatory effector T-cell subsets (proinflammatory Teffs), such as Th1, Th17, and Th22 cells, promote the onset and progression of OA, whereas regulatory T-cell (Treg) subsets inhibit this effect. Tregs and proinflammatory Teffs are both opposing and complementary, creating an immune balance that influences the progression of OA.

This article discusses the intrinsic mechanisms of OA from the perspective of the immune imbalance between Tregs and proinflammatory Teffs, highlighting that the abnormal upregulation of proinflammatory Teffs and the downregulation of Tregs may serve as early disease biomarkers for OA. Therefore, therapeutic strategies aimed at promoting immunoregulation, anti-inflammatory responses, and the expansion of Tregs may represent promising approaches for the treatment of OA. MSCs, owing to their anti-inflammatory and immunomodulatory properties, are considered an ideal option for implementing this novel therapeutic strategy. However, due to the numerous limitations associated with MSCs, such as challenges in transplantation, heterogeneity, and donor sources, the application of MSC-Exos in the treatment of OA may represent a more promising therapeutic approach.
